# Long-Term Effectiveness and Safety of Proactive Therapeutic Drug Monitoring of Infliximab in Paediatric Inflammatory Bowel Disease: A Real-World Study

**DOI:** 10.3390/pharmaceutics16121577

**Published:** 2024-12-10

**Authors:** Susana Clemente Bautista, Óscar Segarra Cantón, Núria Padullés-Zamora, Sonia García García, Marina Álvarez Beltrán, María Larrosa García, Maria Josep Cabañas Poy, Maria Teresa Sanz-Martínez, Ana Vázquez, Maria Queralt Gorgas Torner, Marta Miarons

**Affiliations:** 1 Pharmacy Department, Hospital Universitari Vall d’Hebron, Vall d’Hebron Barcelona Hospital Campus, 08035 Barcelona, Spain; sonia.garciagarcia@vallhebron.cat (S.G.G.); maria.larrosa@vallhebron.cat (M.L.G.); mjosep.cabanas@vallhebron.cat (M.J.C.P.); mariaqueralt.gorgas@vallhebron.cat (M.Q.G.T.); martamiaronsf@gmail.com (M.M.); 2 Paediatric Gastroenterology and Clinical Nutrition Department, Hospital Universitari Vall d’Hebron, Vall d’Hebron Barcelona Hospital Campus, 08035 Barcelona, Spain; oscar.segarra@vallhebron.cat (Ó.S.C.); marina.alvarez@vallhebron.cat (M.Á.B.); 3 Department of Paediatrics, Obstetrics and Gynaecology, Preventive Medicine and Public Health of the Universitat Autònoma de Barcelona, 08193 Bellaterra, Spain; 4 Pharmacy Department, Hospital Universitari de Bellvitge-IDIBELL, Feixa Llarga s/n, L’Hospitalet de Llobregat, 08907 Barcelona, Spain; npadulles@bellvitgehospital.cat; 5 Immunology Department, Hospital Universitari Vall d’Hebron, Vall d’Hebron Barcelona Hospital Campus, 08035 Barcelona, Spain; mariateresa.sanz@vallhebron.cat; 6 Department of Applied Statistics, Universitat Autònoma de Barcelona, Bellaterra, 08193 Barcelona, Spain; ana.vazquez@uab.cat; 7 Pharmacy Department, Consorci Hospitalari de Vic, 08500 Barcelona, Spain

**Keywords:** therapeutic drug monitoring, infliximab, inflammatory bowel diseases, children

## Abstract

Background: This study evaluated the long-term effectiveness and safety of a multidisciplinary early proactive therapeutic drug monitoring (TDM) program combined with Bayesian forecasting for infliximab (IFX) dose adjustment in a real-world dataset of paediatric patients with inflammatory bowel disease (IBD). Methods: A descriptive, ambispective, single-centre study of paediatric patients with IBD who underwent IFX serum concentration measurements between September 2015 and September 2023. The patients received reactive TDM before September 2019 (n = 17) and proactive TDM thereafter (n = 21). We analysed for clinical, biological, and endoscopic remission; treatment failure; hospitalisations; emergency visits; and adverse drug reactions. The IFX doses were adjusted to maintain trough concentrations ≥ 5 µg/mL, with specific targets for proactive TDM. Results: Of the 38 patients, 21 had Crohn’s disease (CD), 16 ulcerative colitis (UC), and 1 undetermined IBD. The mean (standard deviation) IFX trough concentrations were 6.83 (5.66) µg/mL (reactive) and 12.38 (9.24) µg/mL (proactive) (*p* = 0.08). No statistically significant differences between groups were found in remission rates or treatment failure. The proactive group had fewer hospitalisations (14.29% vs. 23.53%; *p* = 0.47) and shorter median hospitalisation days (6 vs. 19; *p* = 0.50), although the difference was not statistically significant. The number of patients with adverse reactions (infusion related reactions and infections) was higher in the proactive group (38.10% vs. 23.53%; *p* = 0.34) but the difference was not significantly different. Conclusions: Proactive TDM showed no significant differences in treatment outcomes compared to reactive TDM. However, the results in both the reactive and proactive TDM groups were not worse than those reported in other studies. Further studies with larger samples are needed to optimize the treatment strategies for pediatric IBD patients.

## 1. Introduction

Biological therapy has revolutionised the treatment of paediatric inflammatory bowel disease (IBD). Infliximab (IFX), a recombinant chimeric immunoglobulin G1 monoclonal antibody, neutralises the biological activity of soluble and membrane-bound tumour necrosis factor-alpha (TNF-α) [1]. IFX was the first anti-tumour necrosis factor (anti-TNF) therapy approved for paediatric use in both Crohn’s disease (CD) and ulcerative colitis (UC) [2].

Despite its effectiveness, IFX therapy poses significant challenges. Approximately 10–30% of patients do not respond to induction therapy, and around 40–50% of initial responders eventually lose responsiveness. Both primary non-response and secondary loss of response are due to low trough concentrations, high titres of antibodies to IFX (ATI), or both. This loss of response is associated with disease flares, hospitalisation, surgical intervention, limited therapeutic options, and a decline in quality of life [3,4,5,6,7,8].

In order to quantify the severity of IBD and to evaluate the responses to different treatments, clinical activity indices (Paediatric Crohn Disease Activity Index (PCDAI) and Paediatric Ulcerative Colitis Activity Index (PUCAI)), endoscopic activity scores (Simple Endoscopic Score for Crohn’s Disease (SES-CD) and Mayo for UC), and different serological and fecal inflammatory biomarkers can be used [9,10,11,12].

Therapeutic drug monitoring (TDM) has emerged as a tool for optimising biological therapy in children with IBD. TDM involves measuring drug concentrations and antibody levels for optimised efficacy and reduced toxicity [2,13]. Despite the widespread use in clinical practice, questions remain about the appropriateness of proactive vs. reactive TDM, timing of monitoring (induction, maintenance, or both), frequency of monitoring (weeks to monitor in induction and maintenance), and concentration thresholds for different phenotypes to achieve clinical, biological, and endoscopic remission [2].

Higher trough serum concentrations (Cmins) have been positively associated with better responses to anti-TNF therapy in both adults and children with IBD, including CD and UC [14,15,16]. The European Crohn’s and Colitis Organisation (ECCO) and the European Society of Paediatric Gastroenterology, Hepatology, and Nutrition (ESPGHAN) guidelines recommend IFX-targeted Cmins ≥25 and ≥15 µg/mL at infusions two (week = 2) and three (week = 6), respectively, and in maintenance a Cmin ≥5 µg/mL for endoscopic healing [3,14]. Specific phenotypes, such as perianal fistulising disease and severe very early onset IBD (VEOIBD) (patients whose age of onset is younger than six years), may require in maintenance a Cmin ≥ 12.7 µg/mL for optimal fistula and mucosa healing [3,17,18,19].

TDM strategies can be categorised as reactive or proactive [2,13,20]. Reactive TDM is used to assess partial response and secondary loss of response, while proactive TDM guides dose individualisation to target appropriate Cmins, potentially reducing the risk of disease relapse, treatment failure, and drug immunogenicity [21,22,23]. The proactive TDM of IFX can also aid in treatment de-escalation [24,25,26,27,28] and optimising monotherapy, avoiding the need to use an immunomodulator, thereby avoiding potential toxicities [29,30]. The preliminary data, primarily from retrospective studies, suggest that proactive TDM is beneficial for patients with IBD [22,23,31]; however, its application in clinical practice remains controversial due to the limited prospective studies [15] and randomised controlled trials (RCTs) [32,33,34,35].

The combination of TDM with a Bayesian approach using population pharmacokinetic (popPK) models has been proposed for enhanced efficacy [20,23,26,34]. However, this model-informed precision dosing (MIPD) requires evidence of improved efficacy, reduced toxicity, or reduced costs to be widely adopted.

This study aimed to evaluate the long-term effectiveness and safety of a multidisciplinary early proactive TDM programme as a tool for IFX dose adjustment in a real-world dataset of paediatric patients with IBD.

## 2. Material and Methods

### 2.1. Study Design

This study was a descriptive, longitudinal, ambispective, single-centre study including paediatric patients (≤18 years old) with IBD who underwent IFX serum concentration measurements between September 2015 and September 2023. The study was conducted under the follow-up of the Paediatric Gastroenterology Department of the Hospital Universitari Vall d’Hebron (HUVH), Spain. Approval for this study was granted by the Clinical Research Ethics Committee of the HUVH (protocol code SCB-INF-2020-01; March 2020), and all patients or their relatives provided written informed consent.

In September 2015, IFX serum concentration measurements were initiated at the HUVH, and by September 2019, proactive TDM utilising MIPD was implemented. The study was divided into two distinct periods based on the policy for IFX dose adjustment: (1) phase 1, which included retrospective data from a 48-month pre-intervention period; (2) phase 2, which included prospective data from a 48-month intervention period. In phase 1, before September 2019, IFX dose adjustments were performed based on TDM and the clinical response (reactive TDM). In phase 2, starting in September 2019, IFX dose adjustments were performed using MIPD during both the induction and maintenance phases (proactive TDM).

To avoid confounding bias, data from the proactive TDM period were excluded for patients who had initially been monitored reactively. This ensured a clear comparison of the efficacy and safety of proactive vs. reactive TDM.

**Figure 1:**
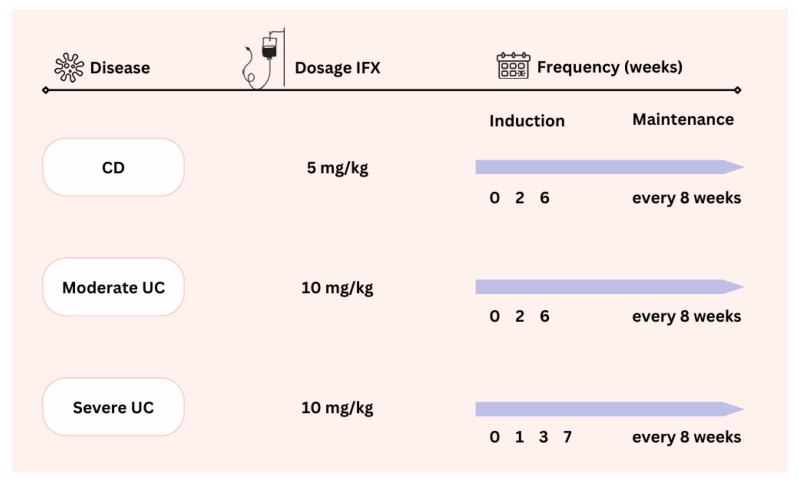
Dosing regimen of infliximab (IFX) according to the types of inflammatory bowel disease. CD = Crohn’s disease; UC = ulcerative colitis.

Figure 1 shows the dosing regimens for intravenous IFX (Remicade^®^ or biosimilar, Inflectra^®^, or Remsima^®^) according to the type of IBD (CD, moderate UC, or severe UC) used in our centre. The UC severity was classified using the Mayo score and PUCAI. Moderate UC was defined by a Mayo score of 2 with a PUCAI < 34 and severe UC by a Mayo score of 3 or a Mayo score of 2 with a PUCAI ≥ 35.

Our TDM proactive protocol included assessments of IFX Cmins at induction (weeks 2 and 6 for CD and moderate UC and weeks 1, 3, and 7 for severe UC) and during maintenance (weeks 14–15 and every six months thereafter). At induction, the target Cmins were >25 µg/mL at weeks 1, 2, and 3 and >15 µg/mL at weeks 6 and 7, and in maintenance the target Cmin range was 5–8 µg/mL. In the case of fistulising CD, including perianal fistula, or severe VEOIBD, the target Cmin range in maintenance was 10–15 µg/mL. The trough target concentrations were based on the recommendations of the ECCO-ESPGHAN guidelines (based mainly on the studies of Clarkston et al., Papamichael et al., and El-Matary et al.) and other previous studies, such as those by Kennedy et al., Yarur et al., and Assa et al. [3,14,15,16,17,18].

### 2.2. Serum Samples and Data Collection

Data from paediatric patients diagnosed with IBD and treated with IFX were collected from routine TDM files (SAP^®^ (Baden-Württemberg, Germany), Silicon (Beirut, Lebanon), and Modulab^®^ (Kortrijk, Belgium) computer systems) and migrated to a common database (Excel^®^, Microsoft 365 MSO, version 2401). Research Electronic Data Capture software (REDCap version 14.2.2, Vanderbilt University, Nashville, TN, USA) was also used to record the data. The patients were coded using correlative numbers, ensuring data dissociation and maintaining confidentiality.

Blood samples were collected from all patients before intravenous infusion to obtain their IFX Cmin and ATI. The following information was recorded for each patient: sex, weight, height, age at diagnosis of IBD (VEOIBD and severe VEOIBD), type of IBD, location or extent and behaviour or severity of the disease, presence of perianal fistulising disease, disease activity (PCDAI for CD and PUCAI for UC), age at the start of IFX administration, concomitant use of immunomodulatory drugs (i.e., thiopurines or methotrexate), extraintestinal manifestations (musculoskeletal, dermatological, haepatopancreatobiliary, ocular, or others), and cigarette smoking. Severe VEOIBD patients were considered those requiring intensified doses and frequencies during maintenance to achieve an IFX Cmin > 10 µg/mL. The disease extent and behaviour were defined according to the Paris Classification [36]. The biological and analytical parameters collected included albumin, faecal calprotectin (FC), erythrocyte sedimentation rate (ESR), serum C-reactive protein (CRP), alanine aminotransferase, aspartate aminotransferase, bilirubin, creatinine, urea, potassium, sodium, and complete blood count (haemoglobin, haematocrit, erythrocytes, platelets, leukocytes, neutrophils, eosinophils, basophils, and monocytes) levels, as well as HLADQA1*05.

### 2.3. Laboratory Tests

The study period overlapped with the use of two different IFX drug-sensitive assays, an enzyme-linked immunosorbent assay (ELISA) with a TRITURUS^®^ analyzer (Grifols, Sant Cugat del Vallès, Spain) until May 2022, followed by chemiluminescence immunoassays (CLIAs) with an IDS-i10 CLIA analyzer (Immunodiagnostic Systems, Boldon, United Kingdom). The ELISA determined the IFX serum concentrations and ATI following the manufacturer’s instructions, using the Promonitor test (Grifols, Sant Cugat del Vallès, Spain) before June 2019 and the Lisa Tracker test (Therediag, Croissy Beaubourg, France) from June 2019 to May 2022. The IFX concentrations are expressed in µg/mL. The lower limit of quantification (LOQ) was 0.035 µg/mL for Promonitor and 0.01 µg/mL for Lisa Tracker. In both cases, the dilution buffer included in the kit was used to dilute those samples with a concentration higher than the upper LOQ (14.4 µg/mL for Promonitor and 20 µg/mL for Lisa Tracker). For Promonitor, ATI are expressed in absorbance units (AU)/mL. The LOQ for ATI was 2 AU/mL and the results are expressed as positive (>2 AU/mL) or negative (<2 AU/mL). In the case of Lisa Tracker, the ATI results are expressed in ng/mL and are detectable (>10 ng/mL) or undetectable (<10 ng/mL).

For the CLIAs, using an i-Traker from Theradiag, the lower LOQ was 0.3 and the upper LOQ was 24 µg/mL, with the dilution for samples exceeding 24 µg/mL. ATI results are categorised as detectable (>10 ng/mL) or undetectable (<10 ng/mL).

ATI determinations were conducted if the IFX concentrations were ≤3 µg/mL (before October 2019, antibodies were measured regardless of the IFX value).

The immunology laboratory validated the correlation between values before transitioning between the different tests (Figures S1 and S2).

### 2.4. Therapeutic Drug Monitoring and Samples

Before September 2019, the IFX Cmins were drawn according to the prescriber’s criteria. From September 2019 onwards, the IFX Cmins were systematically collected before the administration of induction doses (starting in weeks 1 or 2, after the first administration) and maintenance doses (every six months). In case of dose adjustment due to inadequate levels or loss of response, the monitoring times were adapted, during both induction and maintenance, according to the multidisciplinary team’s decision. The blood samples were obtained by venipuncture, collected in a tube with clot activator and gel separator (BD Vacutainer^®^ SST™ II Advance 8.5 mL) for serum determination, and processed within the following 4 h by the HUVH Immunology Laboratory. The samples were centrifuged at 2000× *g* for 10 min and the serum obtained was divided into two aliquots and stored at −80 °C and −20 °C. The serum samples were analysed at the HUVH Immunology Laboratory. The laboratory turn-around time for ELISAs was 15 days and for CLIAs 4 days.

### 2.5. Predictions to Individualise Dosing

The dose prediction started immediately after having an infratherapeutic IFX Cmin, either during induction or maintenance, through MIPD. The observed IFX Cmins before intervention were used to estimate the clearance (CL), inter-compartmental clearance (Q), and central and peripheral distribution volumes (Vc and Vp), using the popPK model previously reported by Fasanmade et al. (2011) and implemented in non-linear mixed-effects modelling (NONMEM) software (version 7.4.3; Icon Development Solutions, Ellicott City, MD, USA) [37]. We calculated the predicted IFX Cmins for various dosing schemes for each patient. After predicting the concentration–time curve using various dosing schemes, we selected the optimal dosing regimen that would maintain the appropriate concentration for each patient. This information, along with biological, clinical, and endoscopic outcomes, was used to draw up a pharmacokinetic report with the patient-adapted dosage recommendation [23,26].

### 2.6. Therapeutic Outcomes

The effectiveness variables were clinical, biological, and endoscopic remission; treatment failure; hospitalisations or emergency visits during treatment; and IBD-related surgery. The follow-up period for IBD-related surgery was extended 24 months beyond IFX discontinuation. The cut-off times to assess remissions and treatment failure were conducted at the end of induction and the end of each year of TDM (weeks 52, 104, 156, and 208).

The disease was considered to be in clinical remission if the disease activity scores were <10. Biological remission was defined as a CRP ≤ 0.5 mg/dL in combination with a decrease in baseline FC (if FC is <250 mg/kg). Lastly, endoscopic remission was considered if there was mucosal healing (Mayo index value ≤ 1) for UC and a SES-CD < 3 for CD [3,38].

The clinical activity scores were determined during routine follow-up visits corresponding to sampling and administration times. The endoscopies were performed according to clinical criteria.

Treatment failure was defined as IFX discontinuation. The reasons for treatment failure included a loss of response despite therapeutic IFX concentrations (pharmacodynamic failure) and serious adverse events that compromised patient safety, including severe infusion-related reactions (SIRRs) or non-reversible ATI. A loss of response was considered as clinical worsening in a patient who previously had a clinical response or remission or an increase in biomarkers (CRP > 0.5 mg/dL and/or FC ≥ 250 mg/dL).

The causes of hospital admissions or emergency visits were classified into IBD-related complications, IBD-related surgery, and IFX-related adverse reactions. IBD-related hospitalisations or emergency visits were defined as any hospitalisation due to disease flare-up (intestinal obstruction, fissure, symptomatic fistula, abscess, or gastrointestinal symptoms secondary to IBD, such as abdominal pain, diarrhoea, constipation, or gastrointestinal bleeding). IBD-related surgical hospitalisation was defined as any hospitalisation due to surgery. The IBD-related surgeries included total or partial bowel resection and ostomy.

Regarding safety, we recorded adverse reactions that could be directly related to IFX (infusion-related reactions (IRRs, infections, and paradoxical psoriasis), as well as the number of emergency department visits or hospitalisations related to these adverse reactions. The adverse reactions were classified as serious (inability to attend school or perform normal daily activity), moderate (sufficient discomfort to reduce or affect normal daily activity), or mild (discomfort was observed but did not affect normal daily activity).

We registered the number of intensifications or de-intensifications performed. The reasons for regimen adjustments included: (1) Cmins outside the established range during both induction and maintenance according to our protocol; (2) high biomarkers (CRP > 0.5 mg/dL and/or FC > 250 mg/kg) and/or clinical non-response (PCDAI and PUCAI > 10); (3) Cmins outside the established range together with high biomarkers and/or clinical non-response; (4) withdrawal of immunosuppression; (5) Cmins outside the established range plus antibody development; (6) changed target levels due a change in the course of the disease.

### 2.7. Statistical Analysis

Descriptive statistics were provided using the median and IQR or mean and standard deviation (SD) for continuous variables and the frequency and percentage for categorical variables. Quantitative variables were tested for a normal distribution using the Shapiro–Wilk test. The continuous normal variables were compared using the t-test and the Wilcoxon test was applied for non-normal variables. The χ^2^ test or likelihood ratio was used for categorical variables, as appropriate.

The effect of the TDM type (proactive vs. reactive) on the cumulative probability of therapeutic outcomes of interest was evaluated using Kaplan–Meier curves for the probability of treatment failure. The curves were compared using the log-rank test. Additionally, a subgroup analysis was conducted to evaluate the effectiveness variables in severe VEOIBD patients.

Proportional hazard models or Cox models were established for the time to treatment failure to determine the effects of different variables that could be associated with therapeutic outcomes. The results are shown as hazard ratios (HRs) and the corresponding confidence intervals for the univariate and multivariate models.

The significance level was set at 0.05 in all tests. All results were obtained with SAS v9.4, SAS Institute, Inc. (Cary, NC, USA).

## 3. Results

### 3.1. Patient Characteristics

A total of 38 patients were included in the study. The patient demographic, clinical, and laboratory characteristics are shown in Table 1 and Table S1. The baseline and disease patient characteristics, as well as laboratory values, were comparable between the proactive and reactive TDM groups. We found no statistically significant differences (*p* > 0.05), except for the number of severe VEOIBDs (*p* = 0.02), as four patients with severe VEOIBD were monitored proactively.

Based on their first IFX Cmin determination, the patients were characterised as having undergone either proactive (21; 55.26%) or reactive (17; 44.74%) TDM. The median (IQR) follow-up periods of the patients were 435 (315–793) days for proactive and 723 (522–1003) days for reactive TDM patients. In reactive TDM, the median (IQR) of the time between IFX therapy initiation and the first IFX Cmin determination was 154 (70–371) days.

### 3.2. Administrations, Trough Concentrations, and Antibodies to IFX

A total of 645 administrations were recorded. The mean (SD) total doses per administration were 9.05 (2.07) and 7.06 (1.63) mg/kg in proactive and reactive TDM, respectively (*p* < 0.01). The frequency rates during maintenance (mean (SD)) were 5.29 (1.72) and 6.99 (1.72) weeks in proactive and reactive TDM, respectively (*p* < 0.01) (Table 2).

Two hundred and sixty-six samples of IFX Cmins were obtained. The mean (SD) Cmin levels during maintenance were 12.38 (9.24) and 6.83 (5.66) µg/mL in the proactive and reactive TDM groups, respectively (*p* = 0.08) (Table 2).

During the induction phase, the percentages of IFX Cmin of patients with proactive TDM achieving target concentrations were 28.57% at weeks 1–3, 50% at weeks 5–7, and 86.66% at the end of induction. During maintenance, target concentrations were achieved in 76.47% and 47.77% of Cmins in the proactive and reactive TDM groups, respectively. Twelve (70.59%), seven (87.50%), and two (100%) patients achieved target concentrations in the proactive TDM group at the end of the first, second, and third year, vs. four (44%), seven (70%), and two (66.67%) patients in the reactive TDM group, respectively.

In the case of severe VEOIBD, the mean (SD) total dose per administration was 10.27 mg/kg (2.37). The frequency (mean [SD]) during induction was 2.70 (1.60) and 3.29 (1.38) weeks during maintenance. Lastly, the mean (SD) Cmin during maintenance was 18.26 (12.77) µg/mL. As shown in Table 2, all four patients with severe VEOIBD were on proactive monitoring.

Regarding immunogenicity, three patients developed ATI, one of whom, with an ATI level of 22.1 ng/mL, did so reversibly after increasing the IFX dosage. The median (range) time to onset of ATI was 282 (14–637) days (Table 2).

### 3.3. Treatment Intervention

A total of 93 out of 266 (35%) patient-adapted dosages and/or frequencies were adjusted after the IFX Cmins were determined (65 intensifications and 28 de-intensifications). In proactive TDM, there were 1.10 patient-adapted regimens per 100 days of TDM during induction and 0.57 during maintenance (Table 3). This rate increased in severe VEOIBD patients, with 1.82 patient-adapted regimens per 100 days of treatment during induction and 0.87 during maintenance (Table 4).

The IFX regimen was maintained without any adjustment during the period that the patient was proactively monitored in 2 out of 21 patients.

The IFX induction regimen was modified in 13 of the 21 (61.90%) patients monitored proactively. Analysing the data by subgroups, the IFX dosing during induction, according to our protocol, was modified in 6 of the 11 patients with CD (54.54%) and 7 of the 10 (70%) with UC.

**Figure 2:**
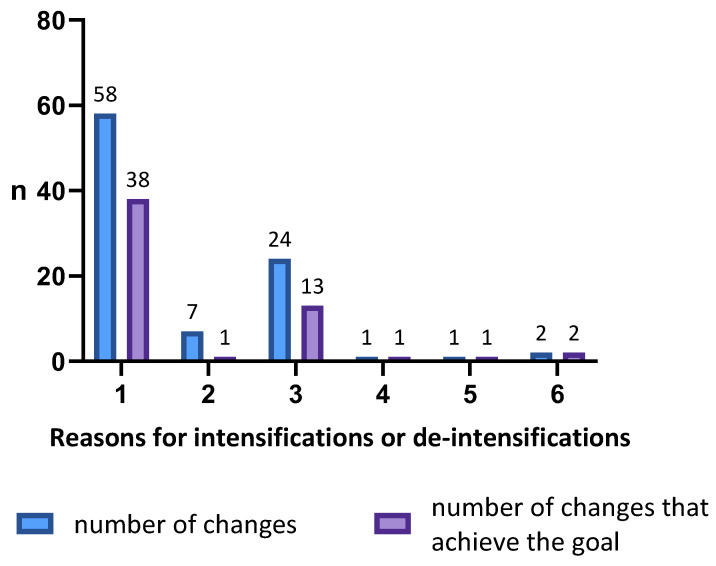
Reasons for intensifications or de-intensifications of infliximab: (**1**) Cmin outside the established range during both induction and maintenance according to the institutional protocol; (**2**) high clinical biomarkers (CRP > 0.5 mg/dL and/or FC > 250 mg/kg) and/or clinical non-response (PCDAI and PUCAI > 10); (**3**) Cmin outside the established range combined with high biomarkers and/or clinical non-response; (**4**) immunosuppression withdrawal; (**5**) Cmin outside the established range plus antibody development; (**6**) change in target levels due to a change in the disease. Cmin = trough concentration; CRP = C-reactive protein; FC = faecal calprotectin; PCDAI = Paediatric Crohn’s Disease Activity Index; PUCAI = Paediatric Ulcerative Colitis Activity Index.

The only reason for de-intensification was a Cmin above the recommended levels. In cases of intensification, the main reason for a change in dosing was a Cmin below the range (62.37%), followed by a Cmin below the range together with high biomarkers and/or clinical non-response (25.81%). The percentage of total treatment interventions that achieved their goal was 59.25% during induction and 60.60% during maintenance. If we analyse this according to monitoring strategy, the percentage of treatment interventions that achieved their goals during induction was 63.16% in proactive TDM vs. 33.33% in reactive TDM and during maintenance, and 61.82% in proactive TDM vs. 54.54% in reactive TDM. Figure 2 shows a visual representation of the reasons for treatment interventions and the number of interventions that achieved the established therapeutic objective.

### 3.4. Efficacy

#### 3.4.1. Treatment Failures and Loss of Response

Of the entire cohort, seven (18.42%) patients experienced treatment failure during follow-up (median (IQR) of 652 {371–965} days), four (19.04%) in the proactive TDM group and three (17.64%) in the reactive TDM group. Two patients (5.26%) experienced primary loss of response, both in the proactive TDM group; five (14.28%) experienced secondary loss of response, two in the proactive vs. three in the reactive TDM group. The causes of primary loss of response were ATI generation with subsequent SIRR in one case and pharmacodynamic failure in the other. Regarding secondary loss of response, one was due to ATI generation and the other four were due to pharmacodynamic causes. There were no treatment failures due to adverse reactions. During the study period, there were losses of follow-up for reasons other than treatment failure, such as the transition from the paediatric to the adult IBD unit (n = 3), moving out of the hospital’s catchment area (n = 1), and a lack of control of extraintestinal symptoms (joint) (n = 1). Notably, there was a decrease in the number of patients over time, as not all patients had prolonged follow-up periods (Table 5).

**Figure 3:**
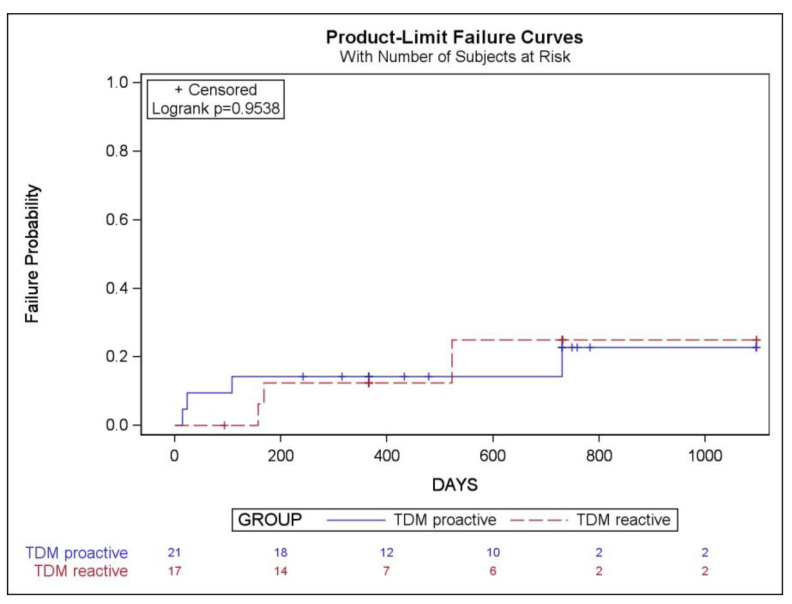
Kaplan–Meier cumulative probability curves for treatment failure with infliximab in children with inflammatory bowel disease undergoing proactive or reactive therapeutic drug monitoring (TDM).

We did not find any statistically significant differences in treatment failures between patients who underwent proactive and reactive TDM (Table 5). The Kaplan–Meier analysis did not demonstrate a significantly lower cumulative probability of treatment failure in patients who underwent proactive compared to reactive TDM (log-rank *p* = 0.95) (Figure 3).

**Figure 4:**
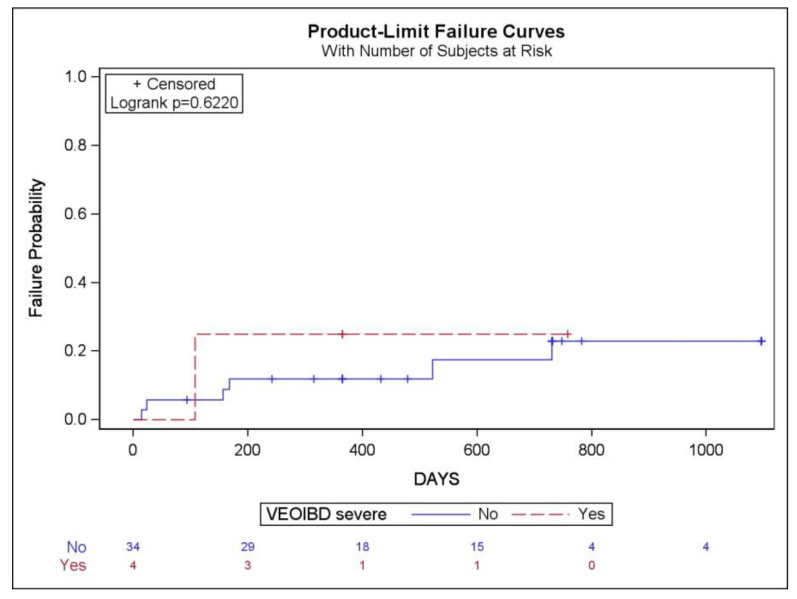
Kaplan–Meier cumulative probability curves for treatment failure with infliximab in children with inflammatory bowel disease according to severe or non-severe very early onset inflammatory bowel disease (VEOIBD).

Regarding treatment failures by type of IBD, comparing the four severe VEOIBD patients with the 34 other children, at week 52, the severe VEOIBD patients had higher treatment failure rates (25% vs. 6.45%; *p* = 0.25) (Table 6). If we analyse the ratio between treatment failures and follow-up times with IFX (years), we obtain a ratio of 0.20 for severe VEOIBD and 0.10 for not severe VEOIBD (0.05 for CD, 0.17 for UC) (Table 6 and Table S2). There is no significant difference in the cumulative probability of treatment failure between patients with severe VEOIBD and those without (log-rank *p* = 0.62) (Figure 4). However, there does seem to be a trend suggesting a higher likelihood of treatment failure in patients with severe VEOIBD.

The Cox regression analysis did not identify the use of proactive TDM (hazard ratio (HR) = 1.045 [95% CI, 0.233–4.68], *p* = 0.95) and severe VEOIBD (HR = 0.589 [0.07–4.94], *p* = 0.63) as variables independently associated with treatment failure. A multivariate Cox analysis was carried out to determine whether there were differences in the risk of treatment failure according to the proactive TDM and VEOIBD variables. None of the variables included in the model were statistically significant (*p* = 0.93 and *p* = 0.63, respectively). The HR for proactive vs. reactive TDM was 0.931 (0.187–4.637) and the HR for severe VEOIBD vs. non-severe IBD was 0.569 (0.058–5.540) in this multivariate Cox analysis.

After the discontinuation of IFX due to treatment failure, two patients underwent IBD-related surgery, one (4.76%) from the proactive TDM group and the other (5.88%) from the reactive TDM group. They were colectomised after discontinuing IFX, with a mean duration (SD) of 14 (5.67) months.

#### 3.4.2. Clinical, Biological, and Endoscopic Remission

We did not find that patients who underwent proactive TDM had a statistically higher probability of clinical and biological remission compared to patients who underwent reactive TDM. However, at the end of the first year, the rate of clinical remission was higher with proactive TDM vs. reactive TDM (84.21% vs. 68.75%; *p* = 0.29) (Table 5).

Regarding clinical and biological remissions by type of IBD, Table 6 shows that severe VEOIBD presented lower remission rates at induction and the end of the first and second years of TDM in comparison with the other patients (0% vs. 73.53%, *p* < 0.01; 50% vs. 64.52%, *p* = 0.58; 0% vs. 82.61%, *p* = 0.07, respectively).

Endoscopic remission could only be confirmed in a few patients, as the endoscopies were performed according to medical decisions and not at defined times. In proactive TDM, during the first year of treatment, five patients underwent endoscopy, only one of whom was in remission (20%); in the second year, of the two endoscopied patients, only one (50%) was in remission. In contrast, at one year of treatment under reactive TDM, four patients underwent endoscopy, three (75%) of whom were in remission; in the second year, of the three endoscopied patients, two were in remission (66%).

In the CD patients, the mean (SD) SES-CD at the start of IFX treatment was 8 (5.28), while at one year of treatment, it was 1 (2.24). At one year of treatment, only four patients underwent endoscopy, of which three (75%) were in endoscopic remission; in the second year, only one patient who had an endoscopy was in remission. For UC, the mean (SD) Mayo score at the start of IFX treatment was 2 (0.62), while at one year of treatment, it was 2 (1.30). At one year of treatment, only five patients underwent endoscopy, and only one (20%) was in endoscopic remission. At the second year, of the four patients who underwent endoscopy, two (50%) were in remission.

For proactive TDM, five of the seven endoscopies were of UC patients, who again achieved worse endoscopic remission outcomes than CD patients.

#### 3.4.3. Hospitalisations and Emergency Visits

Overall, seven (18.42%) patients were hospitalised and four (10.52%) had an emergency visit. Most hospitalisations were due to IBD-related complications (58.33%), followed by IFX-related adverse reactions (33%) and IBD-related surgeries (8.33%). In contrast, the main cause of emergency visits was due to IFX-related adverse reactions (90%), followed by IBD-related complications (10%) (Table 7).

The number of patients with hospital admissions was lower in the proactive than in the reactive TDM group (14.29% vs. 23.53%; *p* = 0.47), and the median (IQR) number of days of admission was lower in the proactive TDM group at 6 (5–14) vs. 19 (7.5–33) days (*p* = 0.50); however, these differences were not statistically significant. There were also no statistically significant difference in the causes of hospitalisation. The ratio of hospitalisations and days of hospitalisation per 1000 days of TDM were lower in proactive vs. reactive TDM at 0.42 and 2.12 vs. 0.54 and 6.22, respectively (Table 7 and Table S3).

Regarding emergency visits, two patients (9.52%) who underwent proactive TDM had eight emergency visits for IFX-related adverse reactions (100%), 80% of which were infections. One of these patients, with VEOIBD, had seven emergency visits. Among the patients undergoing reactive TDM, two (11.76%) had two emergency visits, one due to IBD-related complications and the other to adverse reactions to IFX. There were no significant differences between the two groups (Table S3).

### 3.5. Safety

Infections (60%) were the main reasons for IFX-related adverse reactions, followed by IRR (28.57%) and paradoxical psoriasis (11.43%). Fifty-one per cent of the adverse reactions prevented the patients from attending school or performing normal daily activities, 34.29% had reduced or affected normal daily activity, and 14.29% had normal daily activities. Adverse reactions were the cause of 33% of hospitalisations and 90% of the emergency visits.

The number of patients with adverse reactions was higher in the proactive than in the reactive TDM group at eight (38.10%) vs. four (23.53%), as well as the number of adverse reactions registered with 29 vs. 6. Five (23.81%) patients in the proactive group had an IRR and one (4.76%) had a SIRR. Among the patients who underwent reactive TDM, two (11.76%) had an IRR and none had an SIRR. Regarding infections, five (23.81%) patients experienced them in the proactive TDM group and two (11.76%) in the reactive group (Table 8).

Of the eight patients who presented adverse reactions in the proactive group, three were classified as having severe VEOIBD. Seventy-five per cent of the patients with severe VEOIBD presented adverse reactions. One of these children accounted for 15 of the total 29 adverse reactions reported in the proactive TDM group, with 14 of these 15 adverse reactions being infections (Table 8).

Despite these trends, no significant differences were found between the reactive and proactive TDM groups regarding the adverse-reaction-related variables analysed (Table 8 and Table S4).

## 4. Discussion

We conducted an ambispective single-centre study on the effectiveness of a practice-wide proactive TDM program, aiming to improve the clinical outcomes for paediatric patients with IBD treated with IFX. We observed that the implementation of a proactive TDM program at our institution did not lead to a higher proportion of clinical and biological remissions or a decrease in treatment failure compared to the reactive TDM group, even when reducing hospitalisations. To our knowledge, this is the first comparative study between reactive and proactive TDM that monitors trough concentrations and optimises dosing during induction in proactive TDM for paediatric IBD.

The use of proactive TDM in clinical practice remains controversial due to the limited data from prospective studies and RCTs. The preliminary data, primarily from retrospective studies, indicated that proactive TDM is beneficial in patients with IBD compared with empirical treatment optimisation and/or reactive TDM [2,13]. Papamichael et al. conducted a multicentre study demonstrating that proactive monitoring was associated with better clinical outcomes, including longer drug durability, a reduced need for IBD-related surgery or hospitalisation, and a lower risk of generating ATI or SIRR compared with reactive monitoring [22]. Similarly, Sánchez-Hernández et al. reported that proactive TDM resulted in outcomes comparable to those in the study by Papamichael et al. Sánchez-Hernández et al. compared the proactive TDM cohort with another cohort where dose adjustment was managed with empirical dosing. In the proactive TDM cohort, optimal individualised dosage estimation was addressed using the MIPD approach, as in our study [23]. In 2022, the meta-analysis by Sethi et al. compared TDM vs. empirical dosing and proactive vs. reactive TDM in patients on anti-TNF therapy. Proactive TDM compared to reactive TDM was associated with significant reductions in treatment failure and hospitalisations [21]. In the paediatric population, Lyles et al. showed that proactive anti-TNF TDM improved the rate of steroid-free clinical remission [31].

Three RCTs, TAXIT (Trough Concentration Adapted Infliximab Treatment), TAILORIX (A Study Investigating Tailored Treatment with Infliximab for Active Crohn’s Disease), and PRECISION (Precision Dosing of Infliximab Versus Conventional Dosing of Infliximab), evaluated the proactive TDM of maintenance IFX in IBD patients. As in our study, the superiority of proactive TDM in terms of clinical and biological remission could not be demonstrated either in the TAXIT or TAILORIX clinical trials. However, the inclusion of patients was made after the induction period and the use of 3 µg/mL IFX thresholds. On the other hand, the PRECISION trial used a MIPD to tailor the drug dosing to individual patient’s characteristics (through a Bayesian pharmacokinetic model, incorporating patient data such as gender, body weight, IFX and ATI concentrations, serum CRP, and albumin), demonstrating higher clinical remission in the precision group. An analysis of these trials revealed that the results varied, likely due to differences in study design, population demographics, endpoints, and TDM algorithms [32,33,34].

Multiple prospective exposure–outcome studies, in both adults and children with IBD, and post hoc analyses of RCTs have shown a positive correlation between drug concentrations and favourable therapeutic outcomes [20]. The PANTS (Personalised Anti-TNF Therapy in Crohn’s Disease) study, the largest prospective study of 955 luminal CD patients (≥6 years) treated with IFX, found that low IFX concentrations at week 14 were associated with non-response and ATI occurrence [15].

International guidelines, however, do not provide homogeneous or definitive recommendations for the use of TDM, largely due to the lack of evidence. The American Gastroenterological Association’s 2017 guideline recommended reactive TDM but refrained from making recommendations on proactive TDM [39]. The ECCO-ESPGHAN guidelines, published in 2021, recommend proactive TDM for paediatric CD patients treated with IFX. According to the guidelines, patients should have their first proactive TDM just before the fourth infusion (14 weeks after the initial dose). For patients at risk of accelerated IFX clearance during induction—specifically, children weighing under 30 kg, those with extensive disease, or those with low serum albumin—proactive TDM may be performed as early as the second or third infusion [3]. The Position Statement from the International Association of TDM and Clinical Toxicology describes clinical scenarios where proactive TDM of IFX could be advantageous: firstly, patients starting IFX therapy with high disease activity (acute severe UC), with a disease or phenotype known to require higher IFX exposure (fistulising perianal CD), with a genetic susceptibility for accelerated IFX clearance (carriage of the HLA-DQA1*05 allele), with a young age (below the age of 10 years); secondly, patients in remission considering IFX dose de-escalation and considering the withdrawal of the immunomodulator combination therapy; lastly, patients restarting IFX after a drug holiday [40].

Ongoing prospective trials such as OPTIMIZE (Proactive Infliximab Optimization Using a PK Dashboard in Patients With Crohn’s Disease) (NCT04835506), TITRATE (Induction For Acute Ulcerative Colitis) (NCT03937609), MODIFI (Model-Informed Dose De-Escalation of Infliximab in Patients With Inflammatory Bowel Diseases) (NCT04982172), and REMODEL-CD (Precise Infliximab Exposure and Pharmacodynamic Control to Achieve Deep Remission in Paediatric Crohn’s Disease) (NCT05660746) will provide further knowledge regarding the utility of MIPD in IFX TDM [40,41,42,43]. Actually, there are important efforts to improve the methodological components of MPID, focusing on covariate and concentration-based MIPD and model selection methods [40,44]. Kantasiripitak et al. demonstrates that the use of a model averaging algorithm (MAA) during MIPD had systematically better predictive performance than the model selection algorithm or the single model approach with any model, regardless of the amount of concentration data. This MAA was implemented in the MODIFI study [45]. Kantasiripitak et al. also expanded their work to pediatric patients with IBD [46].

Our proactive TDM group cohort, aiming to achieve trough concentrations ≥5 µg/mL, presented a 19.05% treatment failure rate during a median (IQR) follow-up of 435 (315–793) days, compared to 17.65% over 723 (522–1003) days in the reactive group. Papamichael et al. reported a 13% treatment failure rate in the proactive TDM group vs. 66% in the reactive TDM group during median (IQR) follow-up periods of 876 (547–1204) days and 803 (474–1168) days, respectively [22]. Sánchez-Hernández et al. observed an 18.50% treatment failure rate in the proactive TDM group in comparison with 39.5% in the control group over a three-year follow-up [23]. In our study, severe VEOIBD cases with 25% treatment failure during a median (IQR) follow-up of 376.5 (305–528) days influenced the proactive TDM group’s results. Excluding these cases, our failure rate in proactive and reactive TDM (17.65%) is comparable to Sánchez-Hernández, et al. [19]. In our study, the Kaplan–Meier analysis did not show a higher cumulative probability of treatment failure in severe VEOIBD patients. No significant differences were found in the univariate or multivariate analysis, although there was a trend towards greater treatment failure in severe VEOIBD patients (log-rank *p* = 0.6220). We would like to highlight that the treatment failure rates in reactive TDM were higher in the studies by Papamichael et al. and Sánchez-Hernández et al. compared with our study. Interestingly, and counter-intuitively, there were more treatment failures in Papamichael’s control group, who employed reactive TDM (66%), compared with the study by Sánchez-Hernández et al., in which control group patients were subjected to empirical dosing therapy (39.5%). Moreover, the systematic review and meta-analysis by Sethi et al. [21] compared reactive to proactive TDM in IBD patients on anti-TNF therapy, and found that proactive TDM was associated with a significantly decreased risk of treatment failure (risk ratio (RR), 0.46, 95% CI 0.21 = 0.98, *p* = 0.04).

We also found that the percentages of clinical and biological remission at the end of the induction (57.14% vs. 76.47%) and at week 52 (63.16 vs. 62.50%) were similar in the proactive and reactive TDM groups. In terms of the percentages of clinical and biological remission, our cohort had better results than the PANTS study, even including more severe cases of IBD (fistulous CD, UC, and VEOIBD); the PANTS study reported 42.5% and 39.1% of clinical and biological remission at weeks 14 and 52 in luminal CD patients, respectively [15]. Our results are similar to the TAXIT trial, in which 66% of patients in the clinically based group and 69% in the concentration-based dosing group achieved clinical and biological remission at week 52. However, it should be noted that in our study, higher Cmin values were targeted in comparison with the TAXIT trial [32]. In the TAILORIX trial, the proportion of patients who achieved remission (in this case, corticosteroid-free clinical remission) between 22 and 54 weeks in the dose escalation strategy groups based on clinical symptoms and biomarkers and/or IFX serum concentrations (group 1: increases of 2.5 mg/kg; group 2: increases of 5 to 10 mg/kg) was not higher than the control group, whose adjustment was based on clinical symptoms (dose increase to 10 mg/kg). The remission rates were 33% in group 1, 27% in group 2, and 40% in the control group (*p* = 0.5) [33]. Despite the differences in the methodology of the TAILORIX trial and the definitions of clinical and biological remission, the TAILORIX study achieved lower remission percentages in groups 1 and 2 than in our study. On the other hand, the PRECISION trial demonstrated a higher clinical remission rate in the precision group (88%), in which patients received IFX doses calculated based on MIPD compared with the control group (64%) after one year of treatment [34]. Santacana et al. also provided evidence of the improved short-term efficacy of proactive Bayes-based dosing (MIPD) in a real-world prospective cohort of IBD patients treated with IFX, with rates of clinical remission before and after intervention of 65.7% and 84.3%, respectively [26]. Interestingly, the rates of clinical remission of the precision and intervention groups in the PRECISION trial and the study by Santacana et al. were similar to our proactive TDM group. Lyles et al. performed a retrospective analysis of paediatric IBD, comparing proactive and reactive TDM with anti-TNF, and also found lower clinical remission and clinical and biological remission rates in proactive TDM than in our study at the end of the first year, at 59.2% vs. 84.21% and 42.7% vs. 63.16%, respectively [31].

In our study, the patients undergoing proactive TDM experienced fewer hospitalisations and shorter hospital stays compared with those undergoing reactive TDM. Overall, 14.29% in the proactive group vs. 23.53% in the reactive group were hospitalised, with no statistically significant differences. Even though not statistically significant, this finding is consistent with other studies that suggest that proactive monitoring can reduce the need for acute care interventions. Papamichael et al. reported that 7% of proactive vs. 25% of reactive TDM patients had IBD-related hospitalisations, including surgery [22]. Sánchez-Hernández et al. found that 9.8% of proactive vs. 30.3% of control group patients were hospitalised for IBD, excluding surgery [23]. Our reactive phase results were better but the proactive phase results were worse compared with these studies. The meta-analysis by Sethi et al. indicated that proactive TDM led to significantly reduced hospitalisations compared with reactive TDM [21]. Our median days of IBD-related hospitalisation were lower in the proactive (six days) than in the reactive group (14.5 days). Papamichael et al. also that the number and days of IBD-related hospitalisations were lower in patients undergoing proactive rather than reactive TDM (10 days vs. 40 days, *p* < 0.001; 37 days vs. 189 days, *p* < 0.001, respectively) [18].

After the discontinuation of IFX due to treatment failure, two patients required IBD-related surgery, one (4.76%) in the proactive TDM group and one (5.88%) in the reactive TDM group, within an average of 14 months after discontinuing IFX. In Papamichael et al.’s study, 6% of proactive TDM patients and 19% of reactive TDM patients had IBD-related surgeries within 12 weeks of the last IFX infusion. Nevertheless, the difference in percentages of surgeries between the adult and paediatric populations should not be directly compared due to the shorter time course of the disease in the paediatric population.

Non-reversible ATI cases were detected in 4.76% and 5.88% of proactive and reactive patients, respectively, using a drug-sensitive assay. In the REACH trial, 2.9% of paediatric patients also had positive ATI levels using a drug-sensitive assay [47]. Papamichael et al. found ATI in 9% of proactive TDM patients vs. 28% in reactive TDM, using both drug-sensitive and drug-tolerant assays [22]. Sánchez-Hernández et al. found non-reversible ATI in 4.9% of proactive TDM patients with a drug-sensitive test [23], and the PANTS trial reported 62.8% immunogenicity by week 54 with a drug-tolerant assay [15]. Our results are similar to those of Papamichael et al. and Sánchez-Hernández et al., considering the type of test used.

No treatment failures were attributed to adverse reactions in our study. This is in line with other research, which indicates that while adverse reactions to IFX can occur, they are not the primary cause of treatment failure. Specifically, patients in the proactive TDM group experienced more adverse effects (38.10%) compared with the reactive TDM group (23.53%). Serious adverse reactions occurred in 28.57% of proactive TDM patients vs. 5.88% in the reactive TDM group, although no treatment was discontinued due to adverse effects; serious infections were reported in 19.05% of proactive TDM patients compared with 5.88% in the reactive TDM group. Even though high-dose IFX regimens might be linked to more adverse events, particularly infections, the higher incidence rate in the proactive TDM group may have also been influenced by the inclusion of three patients with severe VEOIBD, with one child accounting for 50% of the adverse events. If these three patients were excluded from the analysis, the differences between the two groups would be diminished. Hendler et al. reported higher rates of serious infections in patients on high-dose IFX, likely due to more severe disease [48]. In the PANTS trial, 17.9% of IFX-treated patients had serious adverse events and 8.8% discontinued treatment due to adverse effects. Serious infections occurred in 4% of patients and the authors concluded that proactive monitoring might potentially reduce serious the adverse events [15]. Unlike in our study, they defined serious adverse events as those requiring hospitalisation or causing significant disability, while our study defined serious events as those preventing normal daily activities. Moreover, the PANTS follow-up was 12 months or until drug withdrawal, while our study had a longer follow-up, making a direct comparison difficult.

In the proactive TDM group of our study, 23.8% of patients experienced IRRs, with 4.8% having an SIRR linked to ATI formation after the second IFX administration. In the reactive TDM group, 11.8% had IRRs and none had SIRRs. Papamichael et al. found that 5% of patients developed SIRRs, mostly in the reactive TDM group (9% vs. 2%) [22]. Sánchez-Hernández et al. reported a lower rate of SIRRs in proactive TDM patients (2.5% vs. 10.4%) [23], and the PANTS trial observed IRRs in 3.2% of patients within 24 h of IFX administration, often associated with ATI [15].

The small sample size did not allow us to observe significant differences in efficacy and safety between the proactive and the reactive TDM groups. In order to guarantee a power rate of 80%, it would be necessary to include a total of 86 patients per group

The dosages used in our paediatric patients were higher than those used in adult studies. Jongsma et al. showed suboptimal trough levels in patients under 10 years of age compared with older patients at the start of maintenance therapy [49]. Paediatric patients have 25–40% lower drug exposure rates compared with adults [50]. Kelsen et al. found that children with CD aged seven or younger had lower IFX response rates and were less likely to continue IFX therapy than older paediatric CD patients [51].

In our paediatric cohort, six patients (15.79%) had VEOIBD and two (5.26%) had infantile-onset IBD. Four of the six VEOIBD patients exhibited more severe behaviour. The disease in younger children is more extensive and requires an optimised treatment regimen. It has been shown that standard IFX regimens and trough levels may not be applicable in this age group and may require more frequent escalation of therapy [2]. In severe VEOIBD, the mean (SD) dosage, frequency, and Cmin in maintenance were 10.49 (2.61) mg/kg, 3.29 (1.38) weeks, and 18.26 (12.77) µg/mL. These results were similar to those described by Adi Eindor-Abarbanel et al. [52] in a retrospective series of 89 VEOIBD patients and the series of four patients with infantile-onset IBD described by Assa et al. [19]. Comparing the four severe VEOIBD patients with the 34 older children, at week 52, the severe VEOIBD patients had higher treatment failure rates (25% vs. 6.45%) and fewer patients in remission (50% vs. 64.52%). These results point to the same trend as the results of Bramuzzo et al.’s study [53], which compared 42 children with VEOIBD to 130 children with IBD. At week 52, fewer VEOIBD children were in remission (15.8% vs. 54.3%, *p* < 0.01) and more had treatment failure (57.89% vs. 30.48%, *p* = 0.03). However, our VEOIBD patients had higher remission rates and lower treatment failure rates compared with Bramuzzo’s study. Therefore, accelerated induction and maintenance dosing with high dosages and low frequencies may have prevented more treatment failures in our VEOIBD series.

Our study had several limitations. First, the small sample size did not allow us to observe significant differences between the proactive and the reactive TDM groups. Second, and in line with the first limitation, the study was conducted in a real-life clinical setting, which while reflecting everyday practice, limits patient inclusion, and may also incorporate variations in clinical decision-making that could influence outcomes. Third, after reviewing the literature, we hypothesised that the lack of observed improvement in treatment failure and remission rates between proactive and reactive TDM in our study may have been because the results from reactive TDM were comparable to those seen in many post-intervention studies, which prevented us from detecting significant differences between groups. Fourth, the retrospective nature of reactive TDM may introduce bias, and a longer follow-up is needed to fully assess the long-term benefits and risks of proactive TDM. Finally, during the study, based on the evolution of laboratory technology, the IFX serum concentrations and ATI were determined using two different ELISA techniques (the Promonitor test, Grifols, Spain; and the Lisa Tracker test, Therediag, France) and CLIAs. However, before changing them, the kits were tested to ensure a good correlation between them. Despite these limitations, the strengths of this study include a long follow-up period and representation of real-life clinical practice at a major referral paediatric IBD centre.

## 5. Conclusions

In our study, reflecting real-life clinical practice, proactive TDM did not demonstrate statistically significant differences in the risks of treatment failure, clinical and biological remission, IBD-related surgery and hospitalisations, ATI, and SIRR in paediatric IBD patients compared to reactive TDM. However, the results in both the reactive and proactive TDM groups were not worse than those reported in other studies. Therefore, the implementation of proactive TDM in clinical practice remains complex and requires further prospective studies with a higher number of patients to establish definitive guidelines and optimise treatment strategies for paediatric IBD patients.

## Figures and Tables

**Table 1:** Baseline characteristics of the patients included in the study.

	Total Cohort (n = 38)	Proactive TDM (n = 21)	Reactive TDM (n = 17)	*p*-Value
Sex; n = men (%)	24 (63.16)	11 (52.38)	13 (76.47)	0.13
Weight (kg) Mean (SD)	37.85 (16.76)	38.67 (19.25)	36.84 (13.58)	0.74
Body Mass Index (kg/m^2^) Median (IQR)	16.71 (15.05–19.23)	16.81 (15.83–19.22)	16.60 (15.05–19.18)	0.62
Age at diagnosis IBD (years) Median (IQR) <2 years; n (%) 2–5 years; n (%) 6–9 years; n (%) 10–17 years; n (%) VEOIBD (<6) years; n (%)	11.68 (6.90–14.26) 2 (5.26) 4 (10.53) 7 (18.42) 25 (65.79) 6 (15.79)	11.05 (6.08–14.62) 2 (9.52) 3 (14.29) 4 (19.05) 12 (57.14) 5 (23.81)	11.92 (11.03–14.20) 0 (0) 1 (5.88) 3 (17.65) 13 (76.47) 1 (5.88)	0.5 0.31
Severe VEOIBD; n (%) Age at diagnosis (years) Median (IQR)	4 (10.53) 1.96 (1.01–2.99)	4 (19.05) 1.96 (1.01–2.99)	0 (0) --	0.02
IBD type; n (%)				0.36
CD	21 (55.26)	11 (52.38)	10 (58.82)	
UC	16 (42.11)	10 (47.62)	6 (35.29)	
Undetermined	1 (2.63)	0	1 (5.88)	
CD				
CD age at diagnosis; n (%)				0.80
A1a	9 (42.86)	5 (45.45)	4 (40.00)	
A1b	12 (57.14)	6 (54.55)	6 (60.00)	
CD Location; n (%)				
L1	5 (23.81)	3 (27.27)	2 (20.00)	0.70
L2	3 (14.29)	1 (9.09)	2 (20.00)	0.47
L3	13 (61.90)	7 (63.64)	6 (60.00)	0.86
L4a	5 (23.81)	3 (27.27)	2 (20.00)	0.70
L4b	1 (4.76)	1 (9.09)	0 (0)	0.25
CD behaviour; n (%)				
B1	19 (90.48)	10 (90.91)	9 (90.00)	0.94
B2	1 (4.76)	0	1 (10.00)	0.21
B3	2 (9.52)	2 (18.18)	0	0.10
*p*	8 (38.10)	6 (54.55)	2 (20.00)	0.10
Growth; n (%)				0.30
G0	17 (80.95)	8 (72.73)	9 (90.00)	
G1	4 (19.05)	3 (27.27)	1 (10.00)	
Perianal fistulising disease; n (%)	6 (28.57)	4 (36.36)	2 (20.00)	0.40
PCDAI Median (IQR)	20 (15–25)	20 (15–27.5)	21.25 (15–25)	0.86
UC				
UC extent; n (%)				0.75
E1	1 (6.25)	1 (10.00)	0	
E2	2 (12.50)	1 (10.00)	1 (16.67)	
E3	2 (12.50)	1 (10.00)	1 (16.67)	
E4	11 (68.75)	7 (70.00)	4 (66.67)	
UC severity; n (%)				0.07
S0	12 (75.00)	9 (90.00)	3 (50.00)	
S1	4 (25.00)	1 (10.00)	3 (50.00)	
PUCAI; Mean (SD)	39.06 (20.10)	36 (20.92)	44.18 (19.34)	0.45
Overall population				
Extraintestinal manifestations Patients; n (%) EIMs, n Median (IQR)	9 (23.68) 11 0 (0–0)	6 (28.57) 8 0 (0–1)	3 (17.64) 3 0 (0–0)	0.43 0.39
Number EIMs by patient; n (%) Patients with 0 EIM Patients with 1 EIM Patients with 2 EIMs	29 (76.32) 7 (18.42) 2 (5.26)	15 (71.43) 4 (19.05) 2 (9.52)	14 (82.35) 3 (17.65) 0 (0)	0.28
Type of EIM, n (% n EIM/patients with EIM) Musculoskeletal Dermatologic Ocular Hepatopancreatobiliary	7 (77.78) 1 (11.11) 1 (11.11) 2 (22.22)	5 (83.33) 1 (16.67) 1 (16.67) 1 (16.67)	2 (66.67) 0 (0) 0 (0) 1 (33.33)	0.58 0.35 0.35 0.58
Age at start of IFX Median (IQR)	13.07 (8.63–15.18)	13.02 (8.63–15.36)	13.11 (11.44–14.71)	0.84
Concurrent medication				
(AZA, 6-MP, MTX); n (%)	35 (92.11)	19 (90.48)	16 (94.12)	0.68
AZA; n (%)	34 (89.47)	19 (90.48)	15 (88.24)	0.82
6-MP; n (%)	1 (2.63)	0	1 (5.88)	0.20
Corticosteroid; n (%)	20 (52.63)	9 (42.86)	11 (64.71)	0.18
Mycophenolate; n (%)	1 (2.63)	0	1 (5.88)	0.20
Tacrolimus; n (%)	1 (2.63)	0	1 (5.88)	0.20
Mesalazine; n (%)	8 (21.05)	6 (28.57)	2 (11.76)	0.20
Smoker; n (%)	1 (2.63)	0	1 (5.88)	0.20
HLA-DQA1*05; n (%) Positive Negative Not determined	11 (28.95) 19 (50.00) 8 (21.05)	5 (23.81) 10 (47.62) 6 (28.57)	6 (35.29) 9 (52.94) 2 (11.76)	0.40
CRP (mg/dL) Median (IQR)	0.98 (0.30–2.91)	1.42 (0.21–3.31)	0.90 (0.48–2.39)	0.95
ESR (mm/h) Median (IQR)	63 (44–110)	55 (42–81)	77 (52–117)	0.20
Faecal Calprotectin (mg/kg) Median (IQR)	821 (382–174)	1150 (520–1740)	534 (299–1844)	0.19
Albumin (g/dL) Median (IQR)	3.80 (3.50–4.10)	3.90 (3.50–4.20)	3.80 (3.50–3.99)	0.46

AZA = azathioprine; CD = Crohn’s disease; CRP = C-reactive protein; ESR = erythrocyte sedimentation rate; EIM = extraintestinal manifestation; 6-MP = 6-mercaptopurine; IQR= interquartile range; MTX = methotrexate; PCDAI = Paediatric Crohn’s Disease Activity Index; PUCAI = Paediatric Ulcerative Colitis Activity Index; TDM = therapeutic drug monitoring; UC = ulcerative colitis; -- = no date.

**Table 2:** Therapeutic drug monitoring outcomes.

	Total Cohort (n = 38)	Proactive TDM (n = 21)	Reactive TDM (n = 17)	*p*-Value
Number of administrations (mean, SD)	645 17.16 (9.51)	349 17 (10.40)	296 17.35 (8.60)	0.91
Dosage of administration (mean, SD) Total (mg/kg) Induction (mg/kg) Maintenance (mg/kg) Maintenance (mg/kg/month)	8.14 (2.12) 7.57 (2.08) 8.31 (2.08) 7.19 (5.12)	9.05 (2.07) 8.34 (2.09) 9.28 (1.98) 9.09 (5.91)	7.06 (1.63) 6.57 (1.60) 7.21 (1.61) 5.05 (2.82)	<0.01 <0.01 <0.01 0.02
Dosage of administration Severe VEOIBD (n = 4) (mean, SD) Total (mg/kg) Induction (mg/kg) Maintenance (mg/kg) Maintenance (mg/kg/month)		10.27 (2.37) 9.72 (1.49) 10.49 (2.61) 16.04 (7.99)		--
Frequency maintenance (weeks) (mean, SD)	6.09 (1.92)	5.29 (1.72)	6.99 (1.72)	<0.01
Frequency maintenance (weeks) Severe VEOIBD (n = 4) (mean, SD)		3.29 (1.38)		--
Number serum samples (Cmin); n (%) Induction Maintenance	266 86 (32.33) 180 (67.67)	211 75 (35.55) 136 (64.45)	55 11 (20.00) 44 (80.00)	
Cmin IFX (µg/mL): induction (mean, SD)	11.48 (12.13)	17.84 (15.10)	7.69 (7.51)	0.06
Cmin IFX (µg/mL): induction (mean, SD) Week 1–3 Week 5–7 Week 12–14	21.28 (15.22) 12.43 (8.67) 10.93 (5.58)	22.36 (15.20) 13.81 (8.00) 11.49 (5.37)	10.02 (14.02) * 3.38 (5.73) * 12.07 (8.21) *	
Cmin IFX (µg/mL): maintenance (mean, SD)	11.05 (8.82)	12.38 (9.24)	6.83 (5.66)	0.08
Cmin IFX (µg/mL): maintenance Severe VEOIBD (n = 4) (median, IQR)		15.70 (10.17–22.2)		--
Patients with ATI (n, %); Irreversible (days between starting IFX and development of ATI) Reversible (days between starting IFX and development of ATI)	3 (7.89%) 2 1	2 (9.52%) 1 (14) 1 (637)	1 (5.88%) 1 (195) 0	

ATI: antibodies to IFX; Cmin: trough concentration; IFX: infliximab; SD: standard deviation; TDM: therapeutic drug monitoring; VEOIBD: very early onset inflammatory bowel disease; * mean (SD) calculated with the number of samples from weeks 1–3 = 2; weeks 5–7 = 3; weeks 12–14 = 3; --: no date.

**Table 3:** Description of optimised posologies in proactive vs. reactive TDM in inflammatory bowel disease (n = 38 patients).

	Optimised Posologies	Intensifications	De-Intensifications	N Cmin	Days of TDM	Optimised Posologies/N Cmin (%)	N Cmin/Days of TDM (%)	Optimised Posologies/Days of TDM (%)
Proactive TDM Induction	21	19	2	75	1909	28	3.9	1.10
Proactive TDM Maintenance	55	32	23	136	9862	40.44	1.38	0.57
Reactive TDM Induction	6	6	0	11	1644	54.55	0.67	0.36
Reactive TDM Maintenance	11	8	3	44	11,381	24.44	0.40	0.10
Total	93	65	28	266	24,796	34.96	1.07	0.38

Cmin = trough concentration; N = number; TDM = therapeutic drug monitoring; days of TDM = total number of days that the patients have been monitored in these period of time.

**Table 4:** Descriptive of optimised posologies in proactive TDM in severe very early onset inflammatory bowel disease (VEOIBD) (n = 4 patients).

	Optimised Posologies	Intensifications	De-Intensifications	N Cmin	Days of TDM	Optimised Posologies/N Cmin (%)	N Cmin/Days of TDM (%)	Optimised Posologies/Days of TDM (%)
Proactive TDM Induction	8	8	0	19	439	42.11	4.33	1.82
Proactive TDM Maintenance	12	5	7	39	1387	30.77	2.81	0.87
Total	20	13	7	58	1826	34.48	3.18	1.10

Cmin = trough concentration; N = number; TDM = therapeutic drug monitoring; days of TDM = total number of days that the patients have been monitored in these period of time.

**Table 5:** Results for remission and treatment failure of proactive vs. reactive TDM at different follow-up times.

	Total Cohort (n = 38)	Proactive TDM (n = 21)	Reactive TDM (n = 17)	*p*-Value
Days of TDM Median (IQR)	24,796 652 (371–965)	11,771 435 (315–793)	13,025 723 (522–1003)	
Nº of patients in w = 0 At the end of Induction Cmin IFX (µg/mL), mean (SD) Clinical Remission; n (%) Biological Remission; n (%) C + B Remission; n (%) Treatment Failure; n (%):	38 10.93 (5.58) 33 (86.84) 26 (68.42) 25 (65.79) 2 (5.26)	21 11.49 (5.37) 18 (85.71) 12 (57.14) 12 (57.14) 2 (9.52):1SIRR/ATI+ 1PD	17 12.07 (8.21) * 15 (88.24) 14 (82.35) 13 (76.47) 0	0.88 0.82 0.11 0.22 0.12
Nº of patients in w = 15 At the end of 1st year (w = 52) Cmin IFX (µg/mL), median (IQR) Clinical Remission; n (%) Biological Remission; n (%) C+B Remission; n (%) Treatment Failure; n (%):	35 8.1 (5.36–10.85) 27 (77.14) 22 (62.86) 22 (62.86) 3 (8.57)	19 9.80 (5.95–10.72) 16 (84.21) 12 (63.16) 12 (63.16) 1 (5.26): PD	16 4.60 (3.62–12.40) 11 (68.75) 10 (62.50) 10 (62.50) 2 (12.50): 1PD + 1ATI	0.28 0.29 0.97 0.97 0.46
Nº of patients in w = 53 At the end of 2nd year (w = 104) Cmin IFX (µg/mL), mean (SD) Clinical Remission; n (%) Biological Remission; n (%) C+B Remission; n (%) Treatment Failure; n (%):	24 8.99 (6.22) 21 (87.50) 20 (83.33) 19 (79.17) 1 (4.17)	11 11.41 (7.39) 9 (81.82) 9 (81.82) 8 (72.73) 0	13 7.04 (4.60) 12 (92.31) 11 (84.62) 11 (84.62) 1 (7.69): 1 PD	0.14 0.45 0.85 0.48 0.26
Nº of patients in w = 105 At the end of 3rd year (w = 156) Cmin IFX (µg/mL), median (IQR) Clinical Remission; n (%) Biological Remission; n (%) C+B Remission; n (%) Treatment Failure; n (%)	9 7.6 (5.27–13.10) 8 (88.89) 7 (77.78) 7 (77.78) 1 (11.11)	3 13.85 (13.10–14.60) 2 (66.67) 1 (33.33) 1 (33.33) 1 (33.33): 1PD	6 5.270 (2.06–7.60) 6 (100) 6 (100) 6 (100) 0	0.08 0.12 0.02 0.02 0.12
Nº of patients in w = 157 At the end of 4th year (w = 208) Cmin IFX (µg/mL), mean (SD) Clinical Remission; n (%) Biological Remission; n (%) C+B Remisission; n (%) Treatment Failure; n (%)	3 5.71 (3.20) 3 (100) 2 (66.67) 2 (66.67) 0	1 9.4 (--) 1 (100) 0 (0) 0 (0) 0	2 3.87 (0.09) 2 (100) 2 (100) 2 (100) 0	-- -- 0.05 0.05 --

ATI = antibodies to infliximab; C+B remission = clinical and biological remission; IQR = interquartile range; PD = pharmacodynamic failure; SIRR = severe infusion-related reaction; TDM = therapeutic drug monitoring; w = week; Cmin IFX = trough concentration of infliximab; days of TDM = total number of days that the patients have been monitored in these period of time; -- = no date; * mean (SD) calculated with a total of 3 samples.

**Table 6:** Results for remission and treatment failure of Crohn’s disease (CD) and ulcerative colitis (UC) vs. severe very early onset inflammatory bowel disease (VEOIBD) at different follow-up times.

	Total Cohort	CD and UC	Severe VEOIBD	*p*-Value
Days of TDM Median (IQR) (days)	24,796 640 (371–926)	22,970 672 (393–926)	1826 376.5 (305–528)	
Nº of patients in w = 0 At the end of Induction Cmin IFX (µg/mL), mean (SD) Clinical Remission; n (%) Biological Remission; n (%) Remission C+B; n (%) Treatment Failure; n (%):	38 10.93 (5.58) 33 (86.84) 26 (68.42) 25 (65.79) 2 (5.26)	34 10.29 (5.16) 30 (88.24) 26 (76.47) 25 (73.53) 2 (5.88): 1 SIRR/ATI + 1PD	4 (10.53) 16.11 (5.42) 3 (75) 0 (0) 0 (0) 0 (0)	0.07 0.47 <0.01 <0.01 0.50
Nº of patients in w = 15 At the end of 1st year (w = 52) Cmin IFX (µg/mL), mean (SD) Clinical Remission; n (%) Biological Remission; n (%) Remission C+B; n (%) Treatment Failure; n (%):	35 8.68 (4.60) 27 (77.14) 22 (62.86) 22 (62.86) 3 (8.57)	31 8.21 (3.89) 25 (80.65) 20 (64.52) 20 (64.52) 2 (6.45): 1 ATI+ 1 PD	4 12.13 (8.68) 2 (50) 2 (50) 2 (50) 1 (25): 1PD	0.52 0.19 0.58 0.58 0.25
Nº of patients in w = 53 At the end of 2nd year (w = 104) Cmin IFX (µg/mL), mean (SD) Clinical Remission; n (%) Biological Remission; n (%) Remission C+B; n (%) Treatment Failure; n (%)	24 8.99 (6.22) 21 (87.50) 20 (83.33) 19 (79.17) 1 (4.17)	23 7.94 (4.49) 21 (91.30) 20 (86.96) 19 (82.61) 1 (4.35): 1 PD	1 26.8 (--) 0 (0) 0 (0) 0 (0) 0 (0)	-- 0.03 0.05 0.07 0.77
Nº of patients in w = 105 At the end of 3rd year (w = 156) Cmin IFX (µg/mL), mean (SD) Clinical Remission; n (%) Biological Remission; n (%) Remission C+B; n (%) Treatment Failure; n (%):	9 8.53 (5.27) 8 (88.89) 7 (77.78) 7 (77.78) 1 (11.11)	9 8.53 (5.27) 8 (88.89) 7 (77.78) 7 (77.78) 1 (11.11): 1 PD	0	-- -- -- --
Nº of patients in w = 157 At the end of 4th year (w = 208) Cmin IFX (µg/mL), mean (SD) Clinical Remission; n (%) Biological Remission; n (%) Remission C+B; n (%) Treatment Failure; n (%):	3 5.71 (3.20) 3 (100) 2 (66.67) 2 (66.67) 0	3 5.71 (3.20) 3(100) 2 (66.67) 2 (66.67) 0 (0)	0	-- -- -- --

ATI = antibodies to infliximab; CD = Crohn’s disease; IQR: interquartile range; PD = pharmacodinamic failure; SIRR = severe infusion-related reaction; UC = ulcerative colitis; VEOIBD = very early onset inflammatory bowel disease; w = week; Cmin IFX = trough concentration of infliximab; days of TDM = total number of days that the patients were monitored in these periods of time; -- = no date.

**Table 7:** Hospital admissions in proactive vs. reactive TDM.

Hospital Admissions	TDM Total	TDM Proactive	TDM Reactive	*p*-Value
Patients; n (%)	38	21 (55.26)	17 (44.74)	
Days of TDM Median (IQR)	24,796 652 (371–965)	11,771 435 (315–793)	13,025 723 (522–1003)	
Hospital admissions, n Patients with hospital admissions; n (%)	12 7 (18.42)	5 3 (14.29)	7 4 (23.53)	0.47
Days of hospital admissions Median (IQR)	13 (5–25)	6 (5–14)	19 (7.5–33)	0.50

IFX = infliximab; IQR = interquartile range; TDM = therapeutic drug monitoring; days of TDM = total number of days that the patients were monitored in these periods of time.

**Table 8:** Adverse reactions of proactive vs. reactive TDM.

Adverse Reaction	TDM Total	TDM Proactive	TDM Reactive	*p*-Value
Patients; n (%)	38	21 (55.26)	17 (44.74)	
AR; n (%)	35	29 (82.86)	6 (17.14)	
Patients with AR; n (%)	12 (31.58)	8 (38.10)	4 (23.53)	0.34
Patients with: IRR as AR bacterial infections as AR viral infections as AR fungal infections as AR paradoxical psoriasis as AR	7 (18.42) 5 (13.16) 1 (2.63) 1 (2.63) 4 (10.53)	5 (23.81) 3 (14.29) 1 (4.76) 1 (4.76) 2 (9.52)	2 (11.76) 2 (11.76) 0 0 2 (11.76)	0.33 0.82 0.27 0.27 0.82

AR = adverse reaction; IRR = infusion-related reaction; TDM = therapeutic drug monitoring.

## Data Availability

The data presented in this study are available on request from the corresponding author due to ethical reasons.

## Supplementary Materials

The following supporting information can be downloaded at: https://www.mdpi.com/article/10.3390/pharmaceutics16121577/s1, Figure S1: Comparison of two commercial enzyme-linked immunosorbent assays (ELISAs) (Promonitor®-Grifols vs. Lisa Tracker®-Therediag) for therapeutic drug monitoring of infliximab (IFX). Pearson’s r: 0.9676; 95% confidence interval: 0.9638–0.9835; R squared: 0.9632; Figure S2: Comparison of an enzyme-linked immunosorbent assay (ELISA) (Lisa Tracker®-Therediag) vs. chemiluminescence immunoassays (CLIAs) (i-tracker®) for therapeutic drug monitoring of infliximab (IFX). Pearson’s r: 0.9610; 95% confidence interval: 0.9399-0.9748; R squared: 0.9235; Table S1: Baseline laboratory parameters of patients included in the study; Table S2: Results regarding remission and treatment failure of Crohn’s disease (CD) vs. ulcerative colitis (UC) vs. severe very-early onset inflammatory bowel disease (VEOIBD) at different follow-up times; Table S3: Hospital admissions and emergency visits in proactive vs. reactive TDM; Table S4: Adverse reactions of proactive vs. reactive TDM.
