# Supplementary material for: Long-Term Effectiveness and Safety of Proactive Therapeutic Drug Monitoring of Infliximab in Paediatric Inflammatory Bowel Disease: A Real-World Study
Source: Pharmaceutics. 2024 Dec 10;16(12):1577. doi: 10.3390/pharmaceutics16121577 (PMC11678755; doi:10.3390/pharmaceutics16121577)
Supplement: Supplementary file 1 [file pharmaceutics-16-01577-s001.zip › pharmaceutics-3329040-supplementary.pdf]

Supplementary Materials: Long-Term Effectiveness and Safety of Proactive Therapeutic Drug Monitoring of Infliximab in Paediatric Inflammatory Bowel Disease: A Real-World Study

Susana Clemente Bautista, Óscar Segarra Cantón, Núria Padullés Zamora, Sonia García García, Marina Álvarez Beltrán, María Larrosa García, Maria Josep Cabañas Poy, Maria Teresa Sanz-Martínez, Ana Vázquez, Maria Queralt Gorgas Torner and Marta Miarons

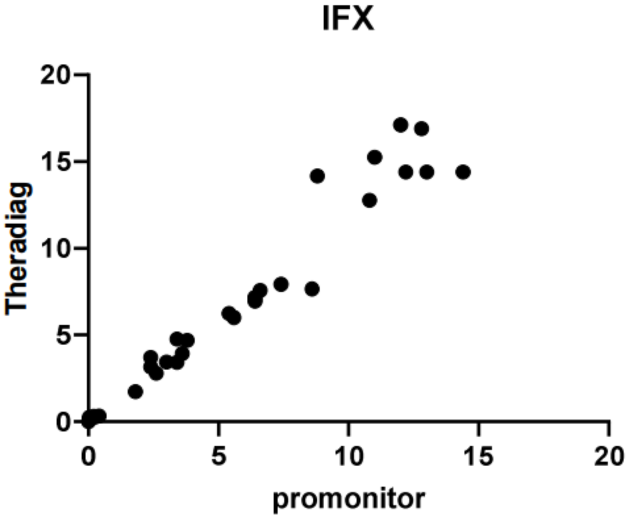

Figure S1. Comparison of two commercial enzyme-linked immunosorbent assays (ELISAs) (Promonitor®-Grifols vs. Lisa Tracker®-Therediag) for therapeutic drug monitoring of infliximab (IFX). Pearson's r: 0.9676; 95% confidence interval: 0.9638–0.9835; R squared: 0.9632.

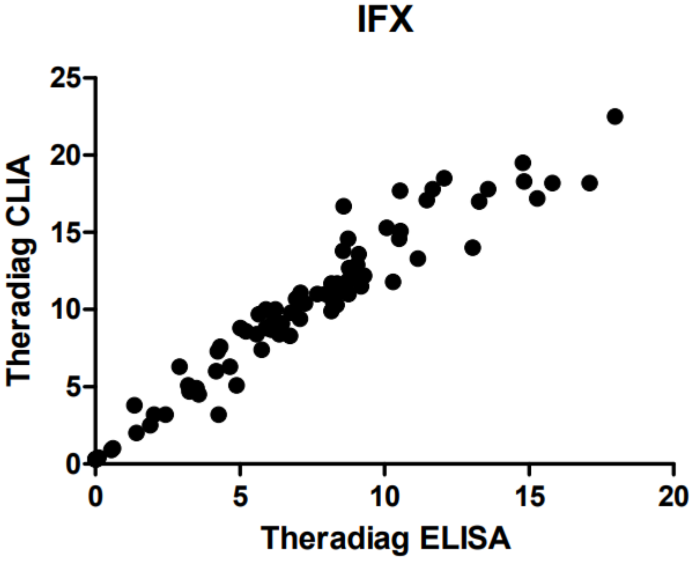

Figure S2. Comparison of an enzyme-linked immunosorbent assay (ELISA) (Lisa Tracker®-Therediag) vs. chemiluminescence immunoassays (CLIAs) (i-tracker®) for therapeutic drug monitoring of infliximab (IFX). Pearson's r: 0.9610; 95% confidence interval: 0.9399-0.9748; R squared: 0.9235.

Table S1. Baseline laboratory parameters of patients included in the study.

Laboratory parameters	Total cohort (n = 38)	Proactive TDM (n = 21)	Reactive TDM (n=17)	*p*-value
ALT (UI/L)				
Median (IQR)	10.50 (6.00-17.00)	12 (7-22)	8 (6-11)	0.09
AST (UI/L)				
Mean (SD)	20.68 (6.98)	21.43 (8.42)	19.76 (4.72)	0.45
Bilirubin (mg/dL)				
Median (IQR)	0.33 (0.22-0.46)	0.33 (0.23-0.46)	0.33 (0.22-0.40)	0.93
Creatinine (mg/dL)				
Median (IQR)	0.44 (0.30-0.63)	0.54 (0.31-0.61)	0.43 (0.29-0.64)	0.61
Urea (mg/dL)				
Median (IQR)	23 (15-31)	24 (16-31)	23 (15-27)	0.75
Potassium (mmol/L)				
Mean (SD)	4.29 (0.39)	4.22 (0.39)	4.37 (0.37)	0.24
Sodium (mmol/L)				
Median (range)	138 (137-139)	138.10 (137.30-139)	137.80 (136.70-139.50)	0.80
Hb (g/dL)				
Median (IQR)	11.20 (9.70-12.30)	11.30 (10.20-12.40)	10.40 (9.10-11.60)	0.17
Haematocrit (%)				
Mean (SD)	34.12 (4.14)	34.78 (3.94)	33.29 (4.34)	0.28
Erythrocytes (x10E^{12}/L)				
Median (IQR)	4.49 (4.16-4.83)	4.49 (4.22-4.83)	4.49 (4.16-4.91)	0.99
Platelets (x10E^{9}/L)				
Mean (SD)	433.97 (145.67)	428.00(135.07)	441.35 (161.75)	0.78
Leucocytes (x10E9/L)				
Median (IQR)	9.71 (7.28-13.99)	9.02 (7.28-14.82)	9.72 (8.33-13.99)	0.52
Neutrophils (x10E9/L)				
Median (IQR)	6.55 (4.70-8.70)	5 (4-8)	6.70 (5.30-10.10)	0.12
Lymphocytes (x10E9/L)				
Median (IQR)	1.80 (1.30-2.80)	2 (1.30-4.10)	1.50 (1.30-4.10)	0.23
Eosinophils (x10E9/L)				
Median (IQR)	0.10 (0-0.30)	0.20 (0.10-0.30)	0.02 (0-0.1)	0.06
Basophils (x10E9/L)				
Median (IQR)	0 (0-0.10)	0 (0-0.10)	0 (0-0.10)	0.83
Monocytes (x10E9/L)				
Median (IQR)	0.75 (0.50-0.90)	0.70 (0.60-1)	0.80 (0.50-0.80)	0.29

ALT = alanine aminotransferase; AST = aspartate aminotransferase; Hb = haemoglobin; IQR = interquartile range.

Table S2. Results regarding remission and treatment failure of Crohn's disease (CD) vs. ulcerative colitis (UC) vs. severe very-early onset inflammatory bowel disease (VEOIBD) at different follow-up times.

Remission/Treatment failure	**Total cohort**	**CD**	**UC**	**severe VEOIBD**	***p*-value**
Days of TDM	24796	14128	8842	1826	
Median (IQR) (days)	640 (371-926)	723 (410-988)	669 (277-852)	376.5 (305-528)	
Nº of patients in w=0	38	19 (50)	15	4	
At the end of Induction					
Cmin IFX (µg/mL), mean (SD)	10.93 (5.58)	8.34 (5)	12.25 (4.88)	16.11 (5.42)	0.07
Clinical Remission; n(%)	33 (86.84)	17 (89.47)	13 (86.67)	3 (75)	0.75
Biological Remission; n(%)	26 (68.42)	16 (84.21)	10 (66.67)	0 (0)	0.03
Remission C+B; n(%)	25 (65.79)	15 (78.95)	10 (66.67)	0 (0)	<0.01
Treatment Failure; n(%):	2 (5.26)	1 (5.26): 1 SIRR/ATI	1 (6.67): 1PD	0 (0)	0.78
Nº of patients in w=15	35	18	13	4	
At the end of 1st year (w=52)					
Cmin IFX (µg/mL), mean (SD)	8.68 (4.60)	7.93 (4.11)	8.54 (3.80)	12.13 (8.68)	0.38
Clinical Remission; n(%)	27 (77.14)	16 (88.89)	9 (69.23)	2 (50)	0.21
Biological Remission; n(%)	22 ((62.86)	14 (77.78)	6 (46.15)	2 (50)	0.18
Remission C+B; n(%)	22 (62.86)	14 (77.78)	6 (46.15)	2 (50)	0.18
Treatment Failure; n(%):	3 (8.57)	1 (5.56): 1 ATI	1 (7.69): 1PD	1 (25): 1PD	0.51
Nº of patients in w= 53<br>At the end of 2nd year (w=104)	24	14	9	1	
Cmin IFX (µg/mL), median (IQR)	6.62 (4.69-12.93)	7.23 (4.32-12.10)	6.00 (5.05-13)	26.80 (26.80-26.80)	0.96
Clinical Remission; n(%)	21 (87.50)	14 (100)	7 (77.78)	0 (0)	0.01
Biological Remission; n(%)	20 (83.33)	13 (92.86)	6 (66.67)	0 (0)	0.09
Remission C+B; n(%)	19 (79.17)	13 (92.86)	9 (81.82)	0 (0)	0.05
Treatment Failure; n(%)	1 (4.17)	0 (0)	1 (11.11): 1PD	0 (0)	0.36
Nº of patients in w=105	9	6	3		
At the end of 3rd year (w=156)					
Cmin IFX (µg/mL), mean (SD)	8.53 (5.27)	8.33 (8.87)	8.66 (4.02)		0.99
Clinical Remission; n(%)	8 (88.89)	6 (66.67)	2 (66.67)	0	0.12
Biological Remission; n(%)	7 (77.78)	5 (83.33)	2 (66.67)		0.58
Remission C+B; n(%)	7 (77.78)	5 (83.83)	2 (66.67)		0.58
Treatment Failure; n(%):	1 (11.11)	0	1 (33.33):1PD		0.12
Nº of patients in w=157	3	3			
At the end of 4th year (w=208)					
Cmin IFX (µg/mL), mean (SD)	5.71 (3.20)	5.71 (3.20)	0		
Clinical Remission; n(%)	3 (100)	3 (100)		0	--
Biological Remission; n(%)	2 (66.67)	2 (66.67)			--
Remission C+B; n(%)	2 (66.67)	2 (66.67)			--
Treatment Failure; n(%):	0	0 (0)			--

ATI = antibodies to infliximab; CD = Crohn's disease; Cmin IFX = trough concentration of infliximab, IQR = interquartile range; PD = pharmacodynamic failure; SIRR = severe infusion-related reactions; UC = ulcerative colitis; TDM = therapeutic drug monitoring, VEOIBD = very early onset inflammatory bowel disease; w = week; days of TDM = total number of days that the patients were monitored in these periods of time.

Table S3. Hospital admissions and emergency visits in proactive vs. reactive TDM.

Hospital admissions/Emergency visits	TDM total	TDM proactive	TDM reactive	*p*-value
Patients; n (%)	38	21 (55.26)	17 (44.74)	
Hospital admissions, n	12	5	7	
Patients with hospital admissions; n (%)	7 (18.42)	3 (14.29)	4 (23.53)	0.47
Number of hospital admissions per patient; n (%):				
Patients with 0 admission	31 (81.57)	18 (85.71)	13 (76.47)	
Patients with 1 admission	3 (7.89)	1 (4.76)	2 (11.76)	0.48
Patients with 2 admissions	3 (7.89)	2 (9.52)	1 (5.88)	
Patients with 3 admissions	1 (2.63)	0	1 (5.88)	
Number of admissions per patient due to IBD-related complications; n (%):				
Patients with 0 admissions	2 (28.57)	0	2 (50)	
Patients with 1 admissions	3 (42.86)	2 (66.67)	1(25)	0.23
Patients with 2 admissions	2 (28.57)	1 (33.33)	1 (25)	
Days of hospitalisation due to IBD-related complications				
Median (IQR)	6 (3-14)	6 (3-14)	14.50 (2-27)	1
Number of admissions per patient due to IBD-related surgery; n (%):				
Patients with 0 admissions	6 (85.71)	3 (100)	3 (75)	0.26
Patients with 1 admissions	1 (14.29)	0	1 (25)	
Number of admissions per patient due to AR related IFX; n (%):				
Patients with 0 admissions	4 (57.14)	2 (66.67)	2 (50)	
Patients with 1 admissions	2 (28.57)	1 (33.33)	1 (25)	0.54
Patients with 2 admissions	1 (14.29)	0	1 (25)	
Emergency visits; n	10	8	2	
Patients with emergency visits; n (%)	4 (10.52)	2 (9.52)	2 (11.76)	
Number of emergency visits per patient; n (%):				
Patients with 0 visits	34 (89.47)	19 (90.48)	15 (88.24)	
Patients with 1 visits	3 (7.89)	1 (4.76)	2 (11.76)	0.41
Patients with 7 visits	1 (2.63)	1 (4.76)	0	
Number of emergency visits per patient due to IBD-related complications				
Patients with 0 visits	3 (75)	2 (100)	1 (50)	0.19
Patients with 1 visits	1 (25)	0 (0)	1 (50)	
Number of emergency visits per patient due to IBD-related surgery				
Patients with 0 visits	4 (100)	2 (100)	2 (100)	--
Patients with 1 visits	0 (0)	0 (0)	0 (0)	
Number of emergency visits per patient due to AR related IFX				
Patients with 0 visits	1 (25)	0 (0)	1 (50)	
Patients with 1 visits	2 (50)	1 (50)	1 (50)	0.25
Patients with 7 visits	1 (25)	1 (50)	0 (0)	

IFX= infliximab; IQR= interquartile range; TDM= therapeutic drug monitoring.

Table S4. Adverse reactions of proactive vs. reactive TDM.

Adverse reactions	TDM total	TDM proactive	TDM reactive	*p*-value
Patients; n (%)	38	21 (55.26)	17 (44.74)	
AR; n (%)	35	29 (82.86)	6 (17.14)	
Patients with AR; n (%)	12 (31.58)	8 (38.10)	4 (23.53)	0.34
Number of AR per patient; n (%):				
Patients with 0 AR	26 (68.42)	13 (61.90)	13 (76.47)	
Patients with 1 AR	6 (15.79)	4 (19.05)	2 (11.76)	
Patients with 2 AR	2 (5.26)	0	2 (11.76)	0.13
Patients with 3 AR	2 (5.26)	2 (9.52)	0	
Patients with 4 AR	1 (2.63)	1 (4.76)	0	
Patients with 15 AR	1 (2.63)	1 (4.76)	0	
Patients with IRR as AR; n (%)				
No	31 (81.58)	16 (76.19)	15 (88.24)	0.33
Yes	7 (18.42)	5 (23.81)	2 (11.76)	
Number of IRR per patient with AR; n (%)				
Patients with 0 IRR	5 (41.66)	3 (37.50)	2 (50)	
Patients with 1 IRR	5 (41.67)	3 (37.50)	2 (50)	0.61
Patients with 2 IRR	1 (8.33)	1 (12.50)	0	
Patients with 3 IRR	1 (8.33)	1 (12.50)	0	
Number of IRR mild per patient; n (%)				
Patients with 0 IRR mild	4 (57.14)	3 (60)	1 (50)	0.81
Patients with 1 IRR mild	3 (42.86)	2 (40)	1 (50)	
Number of IRR moderate per patient; n (%)				
Patients with 0 IRR moderate	4 (57.14)	3 (60)	1 (50)	0.58
Patients with 1 IRR moderate	2 (28.57)	1 (20)	1 (50)	
Patients with 2 IRR moderate	1 (14.29)	1 (20)	0 (0)	
Number of IRR severe per patient; n (%)				
Patients with 0 IRR severe	6 (85.71)	4 (80)	2 (100)	0.39
Patients with 1 IRR severe	1 (14.29)	1 (20)	0 (0)	
Patients with bacterial infections as AR; n (%)				
No	33 (86.84)	18 (85.71)	15 (88.24)	0.82
Yes	5 (13.16)	3 (14.29)	2 (11.76)	
Number of bacterial infections per patient, n (%):				
Patients with 0 bacterial infection	7 (58.33)	5 (62.50)	2 (50)	
Patients with 1 bacterial infection	3 (25)	1 (12.50)	2 (50)	0.38
Patients with 4 bacterial infection	1 (8.33)	1 (12.50)	0	
Patients with 6 bacterial infection	1 (8.33)	1 (12.50)	0	
Number of bacterial infections moderate per patient; n (%):				
Patients with 0 bacterial infection moderate	2 (40)	1 (33.33)	1 (50)	0.55
Patients with 1 bacterial infection moderate	2 (40)	1 (33.33)	1 (50)	
Patients with 2 bacterial infection moderate	1 (20)	1 (33.33)	0	
Number of bacterial infections severe per patient; n (%):				
Patients with 0 bacterial infection severe	1 (20)	0	1 (50)	0.27
Patients with 1 bacterial infection severe	2 (40)	1 (33.33)	1 (50)	
Patients with 2 bacterial infection severe	1 (20)	1 (33.33)	0	
Patients with 5 bacterial infection severe	1 (20)	1 (33.33)	0	
Patients with viral infections as AR; n (%)				
No	37 (97.37)	20 (95.24)	17 (100)	0.27
Yes	1 (2.63)	1 (4.76)	0	
Number of viral infection severe per patient; n (%):				--
Patients with 7 viral infection severe	1 (100)	1 (100)	0	
Patients with fungal infections as AR; n (%)				0.27
No	37 (97.37)	20 (95.24)	17 (100)	

Yes	1 (2.63)	1 (4.76)	0 (0)	
Number of fungal infections mild per patient; n (%):				
Patients with 1 fungal infection mild	1 (100)	1 (100)	0	--
Patients with paradoxical psoriasis as AR; n (%)				
No	34 (89.47)	19 (90.48)	15 (88.24)	0.82
Yes	4 (10.53)	2 (9.52)	2 (11.76)	
Number of paradoxical psoriasis events per patient; n (%):				
Patients with 0 paradoxical psoriasis	8 (66.67)	6 (75)	2 (50)	0.39
Patients with 1 paradoxical psoriasis	4 (33.33)	2 (25)	2 (50)	
Number of paradoxical psoriasis moderate event per patient; n(%)				
Patients with 1 paradoxical psoriasis moderate	4 (100)	2 (100)	2 (100)	--

AR= adverse reaction; IRR= infusion-related reaction; TDM= therapeutic drug monitoring.
